# Prevalence and determinants of self-medication consumption of antibiotics in adults in Iran: a population based cross-sectional study, 2019–2020

**DOI:** 10.3389/fpubh.2024.1502074

**Published:** 2024-12-19

**Authors:** Javad Nazari, Roya Ghafoury, Nahid Chezani-Sharahi, Rahmatollah Moradzadeh, Mobin Naghshbandi

**Affiliations:** ^1^Department of Pediatrics, School of Medicine, Arak University of Medical Sciences, Arak, Iran; ^2^Department of Medicine, Student Research Center, Iran University of Medical Sciences, Tehran, Iran; ^3^Department of Health Services Management, Islamic Azad University, North Tehran Branch, Tehran, Iran; ^4^Department of Epidemiology, Faculty of Health, Arak University of Medical Sciences, Arak, Iran

**Keywords:** self-medication, antibiotics, antibiotics resistance, prevalence, self-consumption

## Abstract

**Introduction:**

The self-medication of antibiotics is a global crisis, posing a significant challenge to healthcare systems worldwide. This study aimed to investigate the frequency of self-medication in the adult population and the factors influencing it.

**Methods:**

This population-based cross-sectional study was conducted in Arak, a city in central Iran, from January 2019 to January 2020. Stratified random sampling was used to determine the recruitment criteria, and a total of 6,692 individuals participated in the study. Self-medication of antibiotics was defined as the self-reported annual consumption of antibiotics, as well as a record of antibiotic use registered in insurance services during the same period. The variables examined in this study included age, gender, educational level, occupational status, insurance coverage, and marital status. All gathered data were analyzed using SPSS version 16.0 and STATA version 16.0 software. *p*-values < 0.05 were considered statistically significant.

**Results:**

The annual prevalence of antibiotic self-medication was 30.3% (*n* = 2,033). Chi-square and Mann–Whitney tests identified a significant correlation between educational level and self-medication practices (*p* = 0.028), while no significant associations were observed with gender, occupation, insurance coverage, or marital status. Logistic regression analysis revealed that female participants were less likely to self-medicate (*p* = 0.027), and both older age and higher levels of education were associated with a reduced likelihood of antibiotic self-medication (*p* = 0.001 and *p* = 0.044, respectively).

**Conclusion:**

Factors such as female gender, older age, and higher education levels are significant determinants affecting antibiotic self-medication.

## Introduction

While drug therapy has significantly benefited humanity, the indiscriminate use of medications can have severe consequences. Self-medication refers to the practice of individuals treating their self-diagnosed symptoms or conditions without consulting a healthcare professional (1). This encompasses reusing prescribed medications for chronic illnesses or similar symptoms, administering one’s medication to family members, prematurely discontinuing treatment after initial symptom relief, using leftover drugs at home, and experimenting with alternative therapies such as herbal remedies or non-professionally recommended drugs (2, 3). Self-medication poses a myriad of risks, including contributing to global health challenges like antimicrobial resistance, a crisis recognized by the WHO. It also increases the likelihood of drug interactions, heightened side effects, accidental poisoning, delays in proper diagnosis and treatment, wastage of time and financial resources, market interference, and a rise in per capita drug consumption. These factors collectively strain public health systems, economies, and societal well-being (4, 5).

The widespread and arbitrary use of antibiotics presents a formidable challenge to healthcare providers globally. Approximately half of all antibiotics are sold over the counter worldwide, with staggering rates of 82% in the Middle East, 75% in Asia, 74% in Africa, 68% in Europe, and 42% in the United States (6–9). This accessibility contributes significantly to the prevalence of self-medication of antibiotics (SMA), particularly in low to middle-income countries. Factors such as economic instability, easy access to medications without prescriptions, inadequate distribution and availability of antibiotics, limited access to professional healthcare, high healthcare costs, long wait times at medical facilities, and patient distrust in medical advice all contribute to this trend (10, 11). The misuse and overuse of antibiotics by the general population have directly fueled the rise of multidrug-resistant bacterial species. These microorganisms pose a critical threat to the effectiveness of current antibiotic treatments, rendering many antibiotics ineffective. Consequently, resistant pathogens lead to difficult-to-treat infections, prolonged illness durations, more complex and costly treatment regimens, increased medical consultations, extended hospital stays, and in severe cases, fatalities (12, 13).

The initial step in tackling the global challenge of antibiotic resistance involves assessing its prevalence and identifying associated risk factors. Armed with this understanding, efforts can be efficiently directed toward developing strategies to curb indiscriminate antibiotic use and mitigate antibiotic resistance (14). Given the limited information on antibiotic self-medication practices in Iran and their potential community-wide impacts, this study was conducted to investigate the prevalence of self-medication with antibiotics (SMA) and its influencing factors among adults in Iran. By examining the extent of SMA and its determinants, this research aims to provide crucial insights into the behaviors and circumstances surrounding antibiotic use in Iran. These findings are essential for informing targeted interventions and policy initiatives aimed at promoting responsible antibiotic practices, safeguarding public health, and preserving the effectiveness of antibiotics in combating infectious diseases.

## Methods

### Study design and setting

This study employed a population-based cross-sectional design conducted in Arak city, located in central Iran, from January 2019 to January 2020. Based on the most recent census data available in 2019, the population of Arak city was approximately 520,944. The study aimed to assess the prevalence and determinants of self-medication with antibiotics (SMA) among adults in Arak.

### Sample size and sampling technique

The sample size of 6,692 participants was determined using statistical calculations to achieve a confidence level of 95% and a margin of error of 1.2%. Stratified random sampling was utilized to ensure demographic representation across key subgroups such as age, gender, and educational level. Stratification was conducted by dividing the population into strata based on these variables, and participants were randomly selected within each stratum proportional to its size in the population.

### Eligibility criteria

Inclusion criteria were as follows:

- Participants had to be Iranian citizens.
- Residents of Arak for a minimum of 3 years.
- Adults aged 18 years and above who were willing to provide informed consent.

Exclusion criteria included individuals who declined to participate or provided incomplete responses.

### Definition of self-medication

Self-medication with antibiotics (SMA) was defined as the self-reported annual consumption of antibiotics that were not corroborated by insurance records during the same period. Insurance records were not used to document SMA directly but served as a benchmark for cross-validation. Antibiotic consumption that did not align with insurance claims was classified as potential SMA.

### Questionnaire development

The questionnaire used for data collection was adapted from validated tools previously employed in similar studies on SMA. Modifications were made to suit the cultural and contextual nuances of the study population. It included sections on demographic and socio-economic variables (age, gender, educational level, occupational status, insurance coverage, and marital status) as well as antibiotic use practices.

### Pilot testing

The adapted questionnaire underwent a pilot test with a subset of 669 participants (10% of the study sample size). Feedback from the pilot study helped refine the questionnaire by identifying ambiguous or irrelevant questions and ensuring cultural appropriateness. Reliability was assessed using Cronbach’s Alpha, which yielded a score of 0.85, indicating good internal consistency.

### Data collection

Data collection was conducted through face-to-face interviews by trained personnel. The interviews were held in public areas, healthcare centers, and community hubs in Arak. Participants were informed about the study objectives, and written informed consent was obtained prior to participation. Privacy and confidentiality were strictly maintained throughout the data collection process. Data were anonymized, securely stored, and used solely for research purposes. To ensure data accuracy, all responses were cross-checked with insurance records where applicable. Discrepancies were resolved through follow-up interviews. The validated dataset provided a robust basis for analyzing the prevalence and determinants of SMA.

### Ethical considerations

The study was approved by the Ethics Committee of Arak University of Medical Sciences (Ethics Code: IR.ARAKMU.REC.1398.207). All procedures adhered to the principles of the Declaration of Helsinki. Participants provided written informed consent, and no personally identifiable information was collected.

### Statistical analysis

Data were analyzed using SPSS version 16.0 and STATA version 16.0 software. Descriptive statistics, including frequencies, means, and standard deviations (SD), were calculated for categorical and continuous variables.

- Association Testing: Chi-square and Mann–Whitney *U* tests were used to compare categorical and continuous variables, respectively.
- Regression Analysis: Univariate logistic regression was performed to identify potential predictors of SMA. Variables with a *p*-value of less than 0.25 were included in a multiple logistic regression model to adjust for confounders and determine independent predictors. Odds ratios (OR) and 95% confidence intervals (CI) were reported.
- Model Validation: The final regression model was assessed for goodness-of-fit and multicollinearity to ensure reliability.

Statistical significance was set at *p* < 0.05 for all tests, and results were interpreted with confidence interval of 95%.

## Results

The descriptive characteristics of the study participants are presented in Table 1. The mean age of participants was 45.03 years (SD = 9.1), with 49.7% being male. A significant portion had attained a diploma as their highest educational level (29.3%), and the majority were employed as housekeepers (45.8%). Insurance coverage was widespread among participants, with 91.1% reporting coverage.

**Table 1:** Descriptive characteristics of the participants.

Variables		*N* (%)	Self-medication consumption of antibiotic		
			No (%)	Yes (%)	*P*-value
Age	Mean (SD)	45.03 (SD = 9.1)			
Gender	Male	3326 (49.7)	2327 (69.9)	1004 (30.1)	0.112
	Female	3366 (50.3)	2437 (72.4)	929 (27.6)	
Education level	Illiterate and preliminary	2234 (33.3)	1653 (47.0)	581 (26.0)	0.028
	Guidance school	1183 (17.7)	771 (65.1)	412 (34.9)	
	High school	220 (3.3)	122 (55.4)	98 (44.5)	
	Diploma	1960 (29.3)	1395 (71.2)	565 (28.8)	
	Higher diploma	340 (5.0)	238 (70.0)	102 (30.0)	
	Bachelor’s degree	613 (9.1)	459 (74.8)	154 (25.2)	
	Master’s degree	142 (2.1)	113 (79.5)	29 (20.5)	
Occupation	Unemployed	103 (1.5)	65 (63.1)	38 (36.9)	0.481
	housekeeper	3068 (45.8)	2224 (72.5)	844 (27.5)	
	Temporary job	1341 (20.0)	885 (66.0)	456 (34.0)	
	Permanent job	1602 (23.9)	1171 (73.1)	431 (26.9)	
	Retired	599 (9.0)	438 (73.1)	161 (26.9)	
Insurance coverage	Yes	6096 (91.1)	4383 (71.9)	1713 (28.1)	0.388
	No	596 (8.9)	379 (63.6)	217 (36.4)	
Marital status	Single	468 (6.9)	393 (83.9)	75 (16.1)	0.088
	Married	6224 (93.1)	4431 (71.2)	1793 (28.8)	

Regarding antibiotic self-medication, the study found an annual prevalence rate of 30.3% (*n* = 2033). Statistical analyses, including chi-square and Mann–Whitney tests, revealed a significant association between educational level and self-medication of antibiotics (*p*-value = 0.028). However, no significant differences were observed by gender, occupation, insurance coverage, or marital status.

Logistic regression analysis results, detailed in Table 2, indicated that female participants exhibited a lower likelihood of self-medicating with antibiotics (*p*-value = 0.027). Furthermore, older age and higher educational attainment were also associated with reduced odds of antibiotic self-medication (*p*-values 0.001 and 0.044, respectively).

**Table 2:** Descriptive characteristics and statistical associations.

Variable	Category	Self-medication (%)	*p*-value
Age (Years)	Continuous (Mean ± SD: 45.03 ± 9.1)	30.3% (2,033 participants)	0.001 (significant)
Gender	Male (49.7%), Female (50.3%)	Male: 30.1%, Female: 27.6%	0.027 (significant)
Education level	Illiterate, Preliminary, Guidance School, High School, Diploma, Higher Diploma, Bachelor’s, Master’s	Varies from 26.0 to 44.5% across categories	0.028 (significant)
Occupation	Unemployed, Housekeeper, Temporary Job, Permanent Job, Retired	Varies from 26.9 to 36.9% across categories	0.481 (not significant)
Insurance coverage	Yes (91.1%), No (8.9%)	Yes: 28.1%, No: 36.4%	0.388 (not significant)
Marital status	Single (6.9%), Married (93.1%)	Single: 16.1%, Married: 28.8%	0.088 (not significant)

## Discussion

This study highlights the significant prevalence of self-medication with antibiotics (SMA) in Arak, Iran, and its contribution to the growing global threat of antimicrobial resistance (AMR). The findings underline the urgent need for practical interventions to curb SMA, including public awareness campaigns to educate communities about the risks associated with AMR and self-prescribing antibiotics. Strict regulatory enforcement is critical to limit over-the-counter access to antibiotics, ensuring they are dispensed only with appropriate prescriptions. Additionally, policymakers should encourage practices such as dispensing antibiotics in precise doses rather than entire packages to reduce leftover medications that often contribute to misuse. Enhancing healthcare accessibility and affordability is also essential to reduce reliance on self-medication. These measures require coordinated efforts from policymakers, healthcare providers, and public health authorities to safeguard the effectiveness of antibiotics and mitigate the burden of SMA on healthcare systems. The self-medication of antibiotics (SMA) poses a global crisis affecting both developed and developing nations. Overuse and misuse of antibiotics have led to widespread antimicrobial resistance among microorganisms, undermining the efficacy of common treatments. While antimicrobial resistance stands as a paramount consequence, SMA also contributes to adverse side effects, drug interactions, and imposes additional economic burdens. Our study focused on investigating the prevalence of SMA and its associated factors among the Iranian adult population. We identified a troublingly high prevalence of SMA and identified several significant factors associated with this practice. These findings underscore the urgent need for health policymakers to reconsider antibiotic stewardship and distribution policies. By limiting public access to over-the-counter antibiotics and promoting responsible use through targeted interventions, we can mitigate the impact of SMA on public health outcomes and preserve the effectiveness of antibiotics for future generations.

In our survey, 30.3% of participants reported practicing SMA over the past year. This rate appears lower compared to previous studies conducted in other regions of Iran. For instance, a study by Heidarifar et al. (15) conducted in Qom, a central province of Iran, reported a SMA prevalence of 57.6% among the general population in 2011. Similarly, research in Shiraz, a southern province, found that approximately 44.5% of patients admitted to primary care centers practiced SMA in 2009 (16). A systematic review from 2015 estimated the overall prevalence of self-medication in Iran to be 53%, with antibiotics being one of the most commonly used over-the-counter medications (17). Over time, there has been a noticeable decline in SMA prevalence in Iran, potentially attributed to factors such as increased public awareness, higher antibiotic costs, and periodic shortages of antibiotics in pharmacies (18, 19). However, compared to other low- to middle-income countries in Asia, Iranian rates remain relatively moderate. For instance, SMA rates were reported as 90.0% in Erbil, Iraq; 84.8% in Karachi, Pakistan; 74.7% in Medina, Saudi Arabia; and 73.2% in Kabul, Afghanistan (20–23). In contrast, many European countries exhibit lower SMA prevalence rates, such as 22% in Lithuania; 20% in Greece; 18.9% in Portugal; and 16% in Romania (24–27). Regarding the sources of antibiotics used in SMA practices in Iran, M. Askarian et al. found that 71.7% of participants obtained antibiotics from pharmacies as over-the-counter medications, while 36% sourced their unprescribed antibiotics from their home drug storage (16). Another study highlighted a high prevalence (82%) of home storage of medications among the Iranian population (28).

Based on our findings, age emerges as a significant factor associated with SMA, with older participants showing lower rates of SMA. This aligns with findings from various studies across different regions. For instance, a Turkish study reported that individuals aged 40–49 years were approximately twice as likely to self-medicate with antibiotics compared to those aged 60–69 years (29). Similarly, Bogale et al. (30) found a decreasing prevalence of SMA with increasing age, with individuals over 60 years having the lowest frequency of SMA. In line with these observations, another study identified age as a significant predictor of SMA, highlighting the highest prevalence among the youngest age group (18–34 years) (26). Likewise, research conducted in Jordan indicated a 1.6-fold higher prevalence of SMA among individuals aged 18–39 years compared to those aged 40–59 years (31). Furthermore, studies focusing on specific demographics, such as mothers, have also noted higher rates of SMA among younger age groups (32). However, contrasting results exist in the literature, with some studies finding no significant association between age and SMA (33, 34).

According to our study findings, women exhibit a lower likelihood of consuming antibiotics on an arbitrary basis compared to men. This observation is consistent with studies conducted in other regions. For instance, Ilhan et al. (29) found that male participants self-medicated with antibiotics 1.24 times more frequently than females. Similarly, research among Portuguese individuals indicated a higher prevalence of antibiotic self-medication among males compared to females (26). However, findings on the association between gender and SMA remain controversial. Some studies have reported no significant difference in the prevalence of SMA between genders (30, 33, 34).

Our study also revealed that participants with higher levels of education exhibit a lower prevalence of SMA. This finding aligns with research conducted by Al-Azzam et al. (31), which similarly demonstrated that individuals with a higher educational background are less likely to engage in SMA practices. Consistent results have been reported across various studies, highlighting the role of higher education in reducing the frequency of SMA (30, 33, 34). Across different countries, there remains a notable lack of public knowledge regarding SMA and its implications for antibiotic resistance (35–38). Common misconceptions persist, such as the belief that antibiotics are effective in treating symptoms like fever, cough, and runny nose, regardless of whether the cause is viral or bacterial (39). Such misunderstandings contribute to inappropriate antibiotic use and the perpetuation of SMA practices. Moreover, previous positive experiences with specific antibiotics may lead individuals to reuse them for subsequent symptoms, despite the potential ineffectiveness or harm (40, 41).

The results of our regression analysis reveal that there is no significant association between SMA and occupational status, insurance coverage, or marital status among our study participants. This finding is consistent with previous studies that have also reported no significant relationships between these variables and the prevalence of SMA (30, 32). Further extensive and rigorous research on the association between independent variables and SMA is needed.

To address the high prevalence of self-medication, it is essential for regulatory bodies to enforce existing legislation strictly and to close loopholes that allow over-the-counter sales of antibiotics. Public health campaigns focusing on antimicrobial resistance and responsible antibiotic use could also reduce the rates of inappropriate consumption. Collaborations between healthcare providers, pharmacies, and policymakers are crucial to develop sustainable interventions.

It is important to note that our study provides valuable insights into the prevalence of SMA and associated sociodemographic factors among a substantial sample of the Iranian adult population (*n* = 6692). To mitigate recall bias, we utilized participants’ insurance records as supplementary data. Given the cross-sectional nature of our study design, it is crucial to acknowledge that we cannot establish a cause-and-effect relationship between SMA and the variables examined. Therefore, future research employing longitudinal strategies, such as cohort studies, is essential to explore and confirm these relationships over time. By conducting more rigorous and longitudinal investigations, we can better understand how factors like occupational status, insurance coverage, and marital status influence antibiotic self-medication practices. This knowledge is vital for developing targeted interventions and policies aimed at promoting appropriate antibiotic use and combating antimicrobial resistance effectively. It is important to note that insurance records may not capture all instances of prescribed antibiotic use, which could lead to some misclassification of self-medication practices.

## Conclusion

This study highlights a significant prevalence of self-medication with antibiotics (SMA) among adults in Arak, Iran, and identifies key demographic factors associated with this behavior. Notably, SMA was found to be higher among certain education and occupation groups, while variables such as marital status and insurance coverage showed no significant associations. These findings emphasize the complexity of SMA and its multifactorial nature, shedding light on patterns that are crucial for designing targeted interventions.

The practice of SMA poses a serious threat to public health, contributing to the growing crisis of antimicrobial resistance (AMR). Our study underscores the urgent need for comprehensive public health strategies, including:

1. Policy Reforms: Stricter enforcement of regulations governing antibiotic sales and dispensing, particularly to prevent over-the-counter access without a prescription.
2. Educational Campaigns: Raising awareness about the risks of SMA and AMR through tailored community-level interventions.
3. Pharmacy Practices: Implementing measures such as dispensing antibiotics in exact doses and encouraging the return of unused medications to pharmacies to reduce antibiotic leftovers.
4. Healthcare Accessibility: Enhancing accessibility and affordability of professional healthcare services to reduce reliance on self-medication practices.

Given the limitations of using insurance records as a benchmark for self-medication and the cross-sectional nature of this study, future research should employ longitudinal designs and explore the effectiveness of specific interventions in reducing SMA. Additionally, comparative studies across different regions of Iran and other countries can provide valuable insights into cultural and systemic factors influencing antibiotic misuse.

Our findings serve as a critical call to action for policymakers, healthcare professionals, and public health authorities to address SMA and safeguard the efficacy of antibiotics in the fight against infectious diseases.

## Data Availability

The raw data supporting the conclusions of this article will be made available by the authors, without undue reservation.
